# Quercetin 3-*O*-Glucuronide from Aglianico Vine Leaves: A Selective Sustainable Recovery and Accumulation Monitoring

**DOI:** 10.3390/foods12142646

**Published:** 2023-07-09

**Authors:** Elena Cioffi, Lara Comune, Simona Piccolella, Mario Buono, Severina Pacifico

**Affiliations:** 1 Department of Engineering, University of Campania “L. Vanvitelli”, I-81031 Aversa, Italy; elena.cioffi@unicampania.it (E.C.); mario.buono@unicampania.it (M.B.); 2 Department of Environmental, Biological and Pharmaceutical Sciences and Technologies, University of Campania “L. Vanvitelli”, I-81100 Caserta, Italy; lara.comune@studenti.unicampania.it (L.C.); severina.pacifico@unicampania.it (S.P.)

**Keywords:** *Vitis vinifera* L. cv. Aglianico, deep eutectic solvents (DES), green extraction, food waste valorization, grape leaves, quercetin glucuronide

## Abstract

In recent years, as part of sustainable development policies, the revaluation of end-of-life products has become more and more widespread. In terms of sustainability, in a scenario of circular economy food sustainability aims, inter alia, at making an effective re-use of natural resources as a starting point for the obtainment of high added-value products. With the aim of promoting the valorization of the wine sector wastes, the present study took into account the leaves of *Vitis vinifera* L. cv. Aglianico from the Campania Region (Italy). The use of deep eutectic solvents as a greener alternative to the most common organic solvents, joint to ultrasound-assisted maceration, and LC-MS tools, allowed us to define for the first time a six-month quantitative variation of flavonol derivatives, and in particular of quercetin 3-*O*-glucuronide, based on the collection time and the leaf height on the grapevine. Results underlined that the influence of abiotic factors, such as exposure to sunlight, which is pivotal in the biosynthesis of such compounds, should be strictly considered for their full recovery.

## 1. Introduction

Most of the current discussion about sustainability in the agri-food sector deals with more effective use of natural resources [1], considering that, regardless of the stage of the production chain, agro-industry generates large amounts of waste and by-products, often not properly disposed of [2], with economic, social, and environmental concerns [3]. While in the past they have often not been properly exploited, except for small purposes within the food chain itself (e.g., feed production), attention has recently increased both to by-products and waste of the food chain and to possible interventions aimed at their valorization. Besides public actions investing in the reduction of food losses or waste, and in response to United Nations 2030 Agenda for Sustainable Development, the scientific community is currently trying to tackle this problem, proposing several strategies to improve the sustainability of the whole food production chain [4]. This includes the exploitation of agri-food waste and by-products (e.g., leaves, skins, seeds, and other inedible parts) in order to obtain high added-value products, such as drugs, nutraceuticals, and cosmeceuticals, thanks to their richness in bioactive compounds. Thus, the concept of food waste recovery moves from the linear “take-make-dispose” model, to become part of the broader concept of circular economy, which can be defined—in a synthetic, but exhaustive way—as a self-sufficient and regenerative system, in which the traditionally considered waste becomes a secondary raw material converted into marketable new products, according to sustainability principles that aim to reduce environmental impacts [5].

Within the agri-food sector, one of the main industrial activities all over the world is represented by winemaking, which takes advantage of about 7 million ha of vineyard surface area, in accordance with the most recent data provided by the International Organization of Vine and Wine in 2021, in which Italy is the major producer, followed by France and Spain [6]. Although the wine industry has been considered environmentally safe if compared to other industrial contexts [7], it should be noted that it is responsible for the production of a huge amount of waste and by-products in a short period of time, among which grape pomace is the most known and widely exploited [8,9,10]. However, even before the vinification begins, grape leaves are the major waste generated in the vineyard, generally disposed of in a landfill or incinerated without a proper estimation of their alternative utilization [11]. Instead, the leaves resulting from defoliation, which takes place mainly in two periods, generally 1–2 weeks after the end of fruit setting to improve grape quality and 2–3 weeks before harvest, are a promising example of a by-product, whose exploitation needs to be pursued as it represents a renewable source of bioactive compounds. 

The valorization of waste and by-products into secondary raw material represents a chance for the industry to strengthen its affordability by converting disposal costs into new profit strategies [12]. In this context, the aim of the present work was to convert the leaves of *Vitis vinifera* L. cv. Aglianico from waste to starting material for a selective and sustainable recovery of polyphenols, which could be introduced in other supply chains, such as those included in the nutraceutical sector. Aglianico is a red grape late variety, probably introduced in the Campania Region (Italy) by the Greeks, and nowadays mainly cultivated in the areas of Avellino and Benevento (Campania Region) and of Potenza (Basilicata Region) [13]. In particular, their exploitation as a source of bioactive molecules with a polyphenolic skeleton [14] has been combined with the use of alternative and more sustainable protocols, in which deep eutectic solvents (DES) have replaced the most common organic solvents, historically dedicated to this purpose, in the ultrasound-assisted extraction (UAE) procedures. DES have been proposed as innovative solvents exhibiting unusual features [15]. They are prepared by mixing two or three low-priced components, such as quaternary ammonium salts (e.g., choline chloride, choline acetate, betaine) and molecules that act as hydrogen bond donors (e.g., amides, organic acids, polyalcohols) [16]. 

In order to valorize leaf waste in the wine production chain, its specialized metabolites should be monitored, also considering that their biosynthesis within the plant tissues is strongly influenced by environmental factors, such as exposure to solar radiation. Thus, a six-month time span has been under evaluation, starting from July (concurrently with agronomic practices) until December (after the harvest has already occurred), collecting the leaves at three different heights on the grapevine.

## 2. Materials and Methods

### 2.1. Leaf Harvesting

The leaves of *Vitis vinifera* L. cv. Aglianico were randomly collected in the vineyard of a winery in Taurasi (Avellino, Italy) (41°00′41.3″ N 14°57′56.7″ E, 371 m a.s.l.) at three heights (upper, middle, and lower) on the vine plant. Six harvesting times were considered, following the agronomic practices of the winery: (1) 22 July 2021; (2) 3 September 2021; (3) 6 October 2021; (4) 28 October 2021; (5) 12 November 2021; (6) 15 December 2021. The harvested leaves were immediately frozen in liquid nitrogen and transferred to the laboratory, where they were lyophilized for 3 days using the FTS-System Flex-dry^TM^ instrument (SP Scientific, Stone Ridge, NY, USA), and then pulverized.

### 2.2. Leaf Extraction and Fractionation

#### 2.2.1. Deep Eutectic Solvents (DES) Preparation

Choline hydrochloride (ChCl; Sigma-Aldrich, Milan, Italy) and citric acid (CA; Sigma-Aldrich, Milan, Italy) were kept in an oven at 45 °C for 1 h before use. They were mixed in a 1:1 molar ratio while heating in a water bath at 80 °C for 30 min. The mixture was constantly stirred until a clear solution was achieved. Then, the solution was diluted with distilled water (20% wt) and stored in a dark glass bottle until use [17].

#### 2.2.2. Extraction and Fractionation Process

The DES mixture, previously prepared, was added to an aliquot (200 mg) of each freeze-dried and powdered sample (1:10, *w*:*w*). After vortexing for 5 min, the samples underwent ultrasound-assisted maceration (UAM) (Ultrasonics^TM^ Bransonic^TM^ M3800-E, Danbury, CT, USA). Three extraction cycles were carried out (30 min each), followed by centrifugation at 10,000× *g* for 20 min in a D3024 DLAB high-speed microcentrifuge (DLAB Scientific Inc., Riverside, CA, USA). 

**Figure 1:**
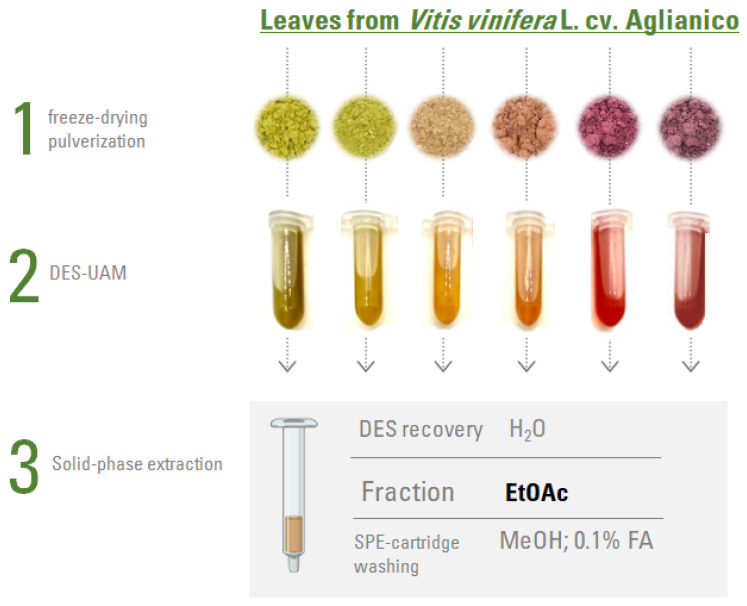
Simplified extraction and fractionation scheme of leaves from *Vitis vinifera* L. cv. Aglianico. (Stage 1: pictures of the pulverized leaves after lyophilization. Stage 2: pictures of the samples obtained after extraction using DES in ultrasound-assisted maceration (UAM). Stage 3: flavonol fractions recovered from solid-phase extraction (SPE).

As the jelly-like supernatants entrapped a residue of the powdered plant matrix, after adding methanol (1:1, *v/v*), a second centrifugation step was performed (10,000× *g*; 10 min). The collected supernatants were dried under vacuum, and subjected to a solid phase extraction, using the Discovery**^®^** DSC-18 SPE tubes (500 mg, 3 mL) (Supelco, Bellefonte, PA, USA). Following the manufacturer’s guidelines, the cartridges were activated with methanol (5 mL) and conditioned with distilled water (15 mL) before use. The sample loading was followed by a washing step using distilled water (10 mL), to remove any interfering substances (including ChCl and CA), then by an eluting step with ethyl acetate (5 mL) to recover the analytes of interest, and, finally, with methanol (0.1% *v/v* of formic acid). This latter step aimed at removing any other potential interferences that could compromise the reliability of further analysis with cartridge reuse. A simplified fractionation scheme is depicted in Figure 1.

### 2.3. Quali-Quantitative Analyses

#### 2.3.1. UV–Vis Spectroscopy

UV–Vis spectra of ethyl acetate fractions from SPE, reconstituted in MeOH (60 µg mL**^−^**^1^), were acquired in the range 200–800 nm by the Cary 100 spectrophotometer (Agilent, Milano, Italy).

#### 2.3.2. UHPLC-ESI-QqTOF-MS Untargeted Profile

In the untargeted approach, UHPLC qualitative profiles were carried out by a NEXERA UHPLC system (Shimadzu, Tokyo, Japan) on a Luna**^®^** Omega Polar C18 (50 × 2.1 mm i.d., 1.6 μm particle size; Phenomenex, Torrance, CA, USA) using as mobile phase water (solvent A) and acetonitrile (solvent B), both acidified with formic acid (0.1 % ***v/v***). The linear gradient started at 5% B, led to 15% in 1 min, kept constant for the following 9 min, then to 45% in the following 2 min, before returning to the initial condition for column re-equilibration. The flow rate was set at 0.5 mL min**^−^**^1^ with a 2 µL injection volume.

Mass spectrometry detection took advantage of the AB SCIEX TripleTOF**^®^** 4600 spectrometer (AB Sciex, Concord, ON, Canada), equipped with a DuoSpray™ ion source, able to operate in both negative and positive electrospray ion mode. The Calibrant Delivery System (CDS) was utilized to automatically calibrate mass-to-charge (*m/z*) values in all scan functions, employing the APCI probe.

TOF-MS experiments were combined with MS/MS in Information Dependent Acquisition (IDA) mode, consisting of a full scan TOF survey (accumulation time 250 ms, 150–1500 Da) and eight IDA MS/MS scans (accumulation time 100 ms, 100–1300 Da). Other source and analyzer parameters were the following: curtain gas 35 psi, nebulizer gas 60 psi, heated gas 60 psi, ion spray voltage −4.5 kV, interface heater temperature 600 °C, declustering potential 70 V, collision Energy (CE) 45 V, CE spread 10 V. The instrument was controlled by Analyst**^®^** TF 1.7 software, while data processing was carried out using PeakView**^®^** software version 2.2.

#### 2.3.3. HPLC-UV-ESI-TQMS Quantitative Analysis 

The targeted quantitative approach was carried out by HPLC 1260 INFINITY II (Agilent Technologies, Santa Clara, CA, USA), coupled with a UV-DAD detector (set at 255, 292, 355 nm) and the AB SCIEX TQ3500 mass spectrometer operating in Multiple Reaction Monitoring (MRM) data acquisition mode. The precursor ion → product ion transitions were *m/z* 477 → 300 (quantification) and 477 → 301 (control) for quercetin 3-*O*-glucuronide, 463 → 300 (quantification) and 463 → 301 (control) for quercetin 3-*O*-hexoside, and 447 → 284 (quantification) and 447 → 285 (control) for kaempferol 3-*O*-hexoside. The amount of each flavonol was calculated, based on the calibration curve of quercetin commercial standard (*y* = 2 × 10^7^ *x* + 1 × 10^7^; 0.0095–0.95 mg mL**^−^**^1^ concentration range) and expressed as mg eq quercetin/g of dried leaves. Data processing was carried out by using the software Sciex OS.

### 2.4. Statistical Analysis

Results were reported as mean values ± standard deviation (SD) deriving from three replicate measurements. Statistical analysis was performed using GraphPad Prism 5 software (GraphPad Software Inc., San Diego, CA, USA). The heatmap was built up using the freely available ClustVis web tool (https://biit.cs.ut.ee/clustvis/, accessed on 16 April 2023).

## 3. Results and Discussion

The selective recovery of flavonol compounds from investigated grape leaves was achieved by optimizing a microextraction using DES in ultrasound-assisted maceration. Based on previous literature data, among several possibilities of combining reagents to prepare DES, the mixture of choline chloride (ChCl) and citric acid (CA) revealed a high selectivity towards flavonoids from several plant materials [18]. Both reagents are registered under the REACH (Registration, Evaluation, Authorization and Restrictions of Chemicals) regulation [19] and are considered generally recognized as safe (GRAS) by the Food and Drug Administration [20]. As the extraction suffers from the close interaction of the flavonols with the components of the eutectic mixture, the removal of the latter is a challenge to be pursued to further use and/or analyze the recovered pure bioactive compounds [21]. DES removal is of great concern, especially when mass spectrometry in negative ion mode is coupled to liquid chromatography techniques for chemical characterization purposes. This is mainly due to the high viscosity of the extracts obtained that prevents the extraction yield calculation, as well as the acidity of the citric acid protons. In fact, it is higher than that of the phenolic and/or alcoholic ones in the (poly)phenol skeleton, causing in the electrospray ionization process a preferential deprotonation of citric acid. Thus, a suppression in the total ion current of the signals occurs, which is attributable to the co-presence of other molecules. For these reasons, the extracts obtained from the leaves of *V. vinifera* cv. Aglianico under study were subsequently fractionated by solid phase extraction (SPE), eluting first with water, to collect DES separately, and then with ethyl acetate (EtOAc), to recover the specialized metabolites of interest.

**Figure 2:**
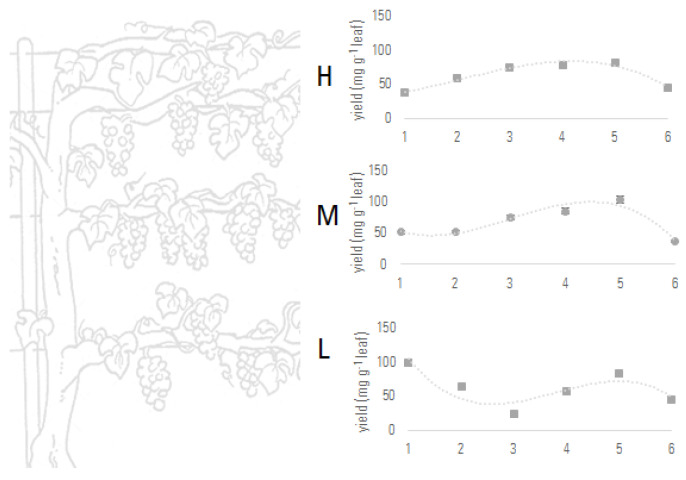
Extraction yields of SPE ethyl acetate fractions under study, obtained from the leaves sampled at six different times (**1**: 22 July 2021; **2**: 3 September 2021; **3**: 6 October 2021; **4**: 28 October 2021; **5**: 12 November 2021; **6**: 15 December 2021) and at three different heights on the grapevine (H: high; M: medium; L: low).

The optimized extraction/fractionation strategy, not time-consuming and expensive, allowed the grape leaves collected at the defined harvesting times to be investigated both qualitatively and quantitatively. This provides, even if only the extraction yield of SPE EtOAc fractions is considered, a first rapid analysis of the variation of the flavonoid content (Figure 2) based on harvesting time and leaf height on plant.

### 3.1. Leaf Extracts’ Qualitative Profile: An Untargeted Approach

In order to obtain preliminary information about the spectral features of the eluted compounds, each EtOAc fraction underwent UV–Vis spectrophotometric analysis. All the fractions showed similar UV profiles in terms of λ_max_ values, differing only in the absorption band intensities, likely ascribable to a different abundance of chemical constituents. In Figure S1 a representative UV–Vis spectrum is reported.

The recorded spectra showed two main absorption bands at 260 and 360 nm, suggesting the occurrence of flavonols. In fact, UV–Vis spectra of these metabolites, like those of flavones, are characterized by two absorption maxima in the region between 240 and 280 nm (the so-called band II) and in the range of 300–380 nm (band I). Band II has been ascribed to the benzoyl system of ring A, while band I refers to the absorption of the cinnamoyl moiety. Therefore, the latter allows the two flavonoid sub-classes to be discriminated. In fact, for flavones, λ_max_ is between 304 and 350 nm, while for flavonols it is found in the range of 352–385 nm. Recently, a flavonol has been isolated and chemically characterized from cv. Greco di Tufo vine leaves, whose spectrum appeared almost superimposable to that reported in Figure S1, showing two absorption bands at 257 and 357 nm, in accordance with the presence of quercetin 3-*O*-glucuronide [22]. The compound is well known for its nutraceutical potential, which relies in its capability to boost insulin signaling and inflammation (similar to the aglycone quercetin), and to inhibit Aβ aggregation in vitro [23]. Anti-inflammatory, antioxidant, moisturizing, and anti-melanogenesis properties have been also described in human keratinocytes via activation of NF-κB e AP-1 pathways [24]. 

**Figure 3:**
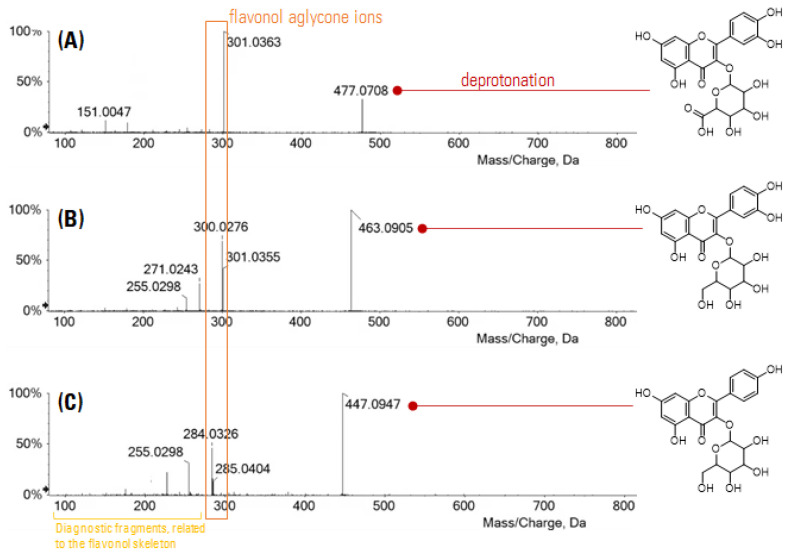
TOF-MS/MS spectra of the detected flavonols: (**A**) quercetin 3-*O*-glucuronide; (**B**) quercetin 3-*O*-hexoside; (**C**) kaempferol 3-*O*-hexoside.

Thus, the two steps DES-UAM-based extraction/SPE-based fractionation appeared to provide semipurified fractions enriched in quercetin 3-*O*-glucuronide. To further confirm the presence of the compound, the UHPLC-HRMS analyses allowed us to clearly detect it, beyond other two flavonols, quercetin 3-*O*-hexoside and kaempferol 3-*O*-hexoside, as major constituents of the investigated samples. In Figure 3 the TOF-MS/MS spectra of the three compounds are reported.

The mass fragmentation of the ion at *m/z* 477.0708 (C_21_H_18_O_13_) gave rise to the base peak at *m/z* 301.0363, corresponding to the deprotonated quercetin aglycone ion, besides low-intensity (<10%) fragments at *m/z* 283.0247 ([aglycone-H-H_2_O]^−^), 273.0406 ([aglycone-H-CO]^−^), 255.0303 ([aglycone-H-CO-H_2_O]^−^), 178.0988 e 151.0047 (ring A) (Figure 3A). These latter were also detected in the spectrum of the hexosyl derivative, although it was mainly characterized by a neutral loss of 162/163 Da from the molecular ion at *m/z* 463.0905 (C_21_H_20_O_12_) that was responsible for the formation of the deprotonated aglycone ion (at *m/z* 301.0355) and its corresponding radical anion (at *m/z* 300.0276). Their intensity ratio suggested that glycosylation occurred at C-3 position (Figure 3B). Similar considerations concerned the last flavonol, tentatively identified as kaempferol 3-*O*-hexoside (C_21_H_20_O_11_) (Figure 3C). Based on HR-MS/MS spectra, the fragmentation schemes were drawn for the investigated compounds, herein reported as Supplementary Materials (Figures S2 and S3).

### 3.2. Leaf Extracts Quantitative Profile 

The accumulation of bioactive flavonols, in particular, quercetin 3-*O*-glucuronide, in the leaf matrix was monitored at the six harvesting times. To this aim, each EtOAc fraction deriving from the SPE procedure underwent quantitative analysis by means of HPLC-UV/DAD and HPLC-MS detection in MRM mode. The flavonol amount was expressed as mg eq quercetin/g of dried leaf, based on the calibration curve of the pure commercial standard (Figure S4). 

**Figure 4:**
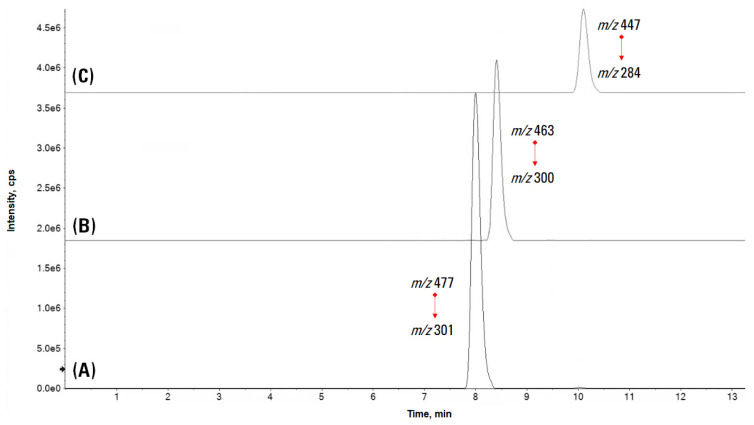
HPLC-MS chromatograms in MRM mode of: (**A**) quercetin 3-*O*-glucuronide; (**B**) quercetin 3-*O*-hexoside; (**C**) kaempferol 3-*O*-hexoside.

The multiple reaction monitoring (MRM) mode was chosen for data acquisition, due to its higher sensitivity, when compared to other MS techniques. Precursor ion → product ion transitions for each analyte and for the standard quercetin were selected based on previously acquired MS/MS spectra. HPLC-MS chromatograms in MRM mode of each flavonol are depicted in Figure 4.

**Figure 5:**
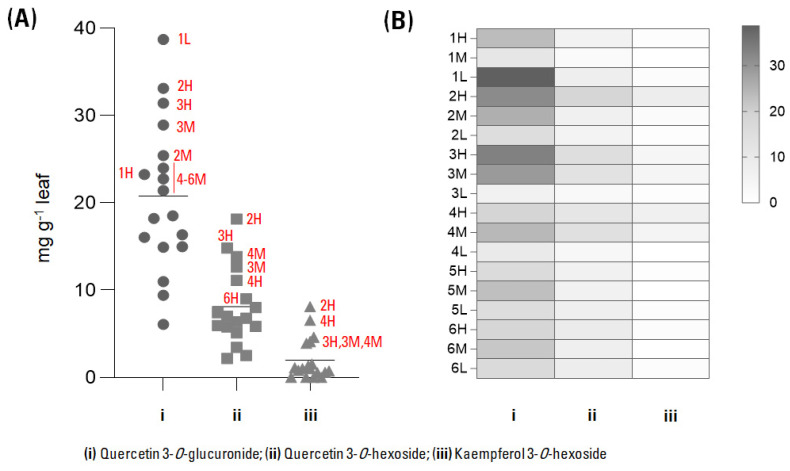
Results of the quantitative analysis, expressed as: (**A**) dot plot, representing mg eq quercetin/g of leaf calculated for the three flavonols; (**B**) heatmap. Leaves were collected at six harvesting times (**1**: 22.07.2021; **2**: 03.09.2021; **3**: 06.10.2021; **4**: 28.10.2021; **5**: 12.11.2021; **6**: 15.12.2021) and at three heights on the grapevine (H = high, M = medium, L = low).

**Figure 6:**
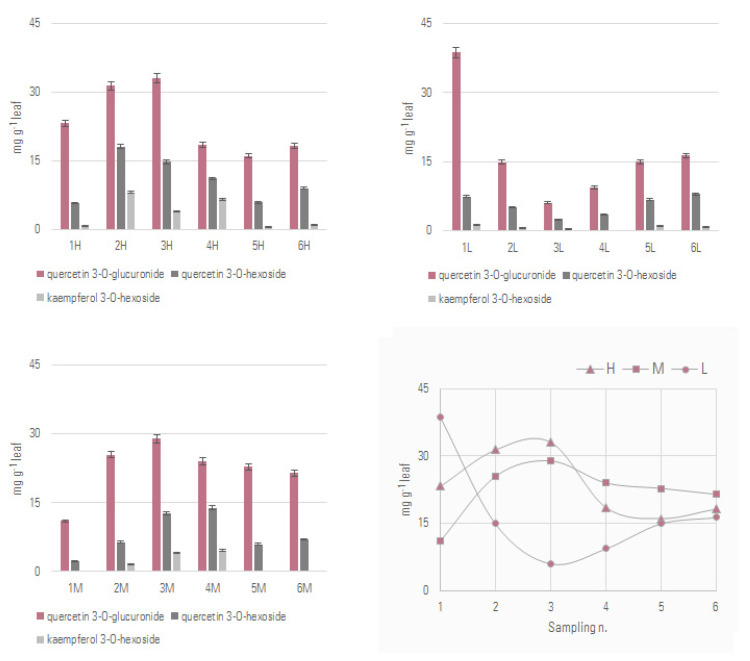
Results of the quantitative analysis, expressed as mg eq quercetin/g of leaf in the investigated samples, grouped based on the leaf heights on the plant (H = high, M = medium, L = low). In the grey box the trend over time of quercetin 3-*O*-glucuronide is reported (harvesting time **1**: 22.07.2021; **2**: 03.09.2021; **3**: 06.10.2021; **4**: 28.10.2021; **5**: 12.11.2021; **6**: 15.12.2021).

The results of the quantitative analysis are reported in Figure 5 and Figure 6, related to leaves collected at the three heights on the grapevine and at the six collection times.

Optimizing the extraction processes could represent the beginning of an integrated production of bioactive substances from residual components of the supply chain for the formulation of new bio-based nutraceutical products. The knowledge of the relative content of the bioactive metabolite opens up new usage scenarios that take into account plant physiology and the specific location of an organ at the plant level. In fact, several factors influence the content of specialized metabolites in a plant: some are intrinsic to the plant itself (endogenous or genetic factors), while others depend on the environment in which the plant grows (exogenous or environmental factors and biotic factors), others concern the collection time, and the preparation and storage of the plant or its organs [25]. In particular, abiotic stress strongly influences the development and the ability to synthesize specialized metabolites, not only in the case of wild plants but also of those cultivated, being the accumulation of such molecules also organ-specific. 

The most representative flavonol in the investigated samples was the glucuronidated derivative of quercetin, followed by the hexosyl derivative and the kaempferol 3-*O*-hexoside. These data were in line with the chemical composition of leaves from the same cultivar, despite being extracted by microwave-assisted extraction, whose HPLC chromatograms were dominated by quercetin glycosylated derivatives, followed by kaempferol ones [26]. 

The quantitative variations appeared dependent not only on the harvest time but also on the height of the sampled leaf. In particular, in the grey box in Figure 6 the changes in the accumulation of quercetin 3-*O*-glucuronide are shown as a function of the two variables considered. Its accumulation in the highest leaves on the shoots increased from July (sampling n. 1) until early October (sampling n. 3), when it reached its maximum, equal to 31.40 mg/g of dried leaf, and then almost halved with the full ripeness of the bunches (sampling n. 4), which is late for cv. Aglianico grapes in the Campania Region, if compared to other red grapes, generally between the second half of October and the first ten days of November [27]. After that, its amount appeared constant (samplings n. 5 and 6). A similar trend was observed for medium-height leaves, whereas for the lowest ones the behavior was the opposite, with a maximum concentration in July 2021, which decreased rapidly as early as September, before reaching the minimum value (equal to 6.07 mg/g of dried leaf) in early October. It is reasonably due to defoliation practices commonly applied till the end of summer to improve both productivity and quality of grapes, which have direct consequences in microclimate and shadow-light ratio changes [28].

Although the nature of flavonols in vine leaves has been previously documented, little data is available to date regarding their fluctuations. Indeed, it has been shown that the concentration of kaempferol 3-*O*-glucoside and its aglycone differed significantly depending on the age of the leaves in the Pinot Noir cultivar [29]. More recently, a study focused on the Cabernet Sauvignon cultivar showed how the regulation of the biosynthesis of some flavonol glycosides in the leaves differed significantly. In detail, a strong positive correlation was found between the amounts of quercetin rutinoside and glucuronide, while similar levels of quercetin galactoside and glucoside, and of kaempferol glucoside and glucuronide, which accumulated more during leaf aging, have suggested a common regulation. Seasonality has also been indicated as an important factor influencing the levels and composition of flavonols. Therefore, their profile was influenced by both age and season: older leaves showed a lower content of quercetin glucuronide and a higher ratio of glucosyl derivative and kaempferol glucuronide [30].

Climatic changes, including light exposure, temperature fluctuation, and rainfall can be counted among the main environmental factors responsible for an increased or decreased rate in the biosynthesis of (poly)phenols. Indeed, different light exposures have different consequences. It has been shown that plant shading decreases the flavonoid content in the leaves. This outcome aligns well with the function these molecules serve in safeguarding plant tissues against UV light. Extreme weather conditions with prolonged droughts and heavy rains can also seriously affect the physiology of the vine [31]. Plant tissues accumulate flavonoids when exposure to UV-B rays increases, through the upregulation of the gene expression of the enzyme flavonol synthase (FLS) by UV-B radiation. Moreover, the flavonoid that best correlates with exposure to UV-B rays is represented by quercetin 3-*O*-glucuronide [32]. In this regard, there would seem to be a positive correlation between the total antioxidant capacity of the leaf and exposure to UV light. Vine leaves with limited stomatal conductance increase photorespiration, and hydrogen peroxide from photorespiration could serve as a messenger molecule to trigger increased antioxidant defense [33].

## 4. Conclusions

The research work presented herein was aimed at valorizing the leaves of *Vitis vinifera* L. cv. Aglianico, the less valued waste in the vineyard resulting from the defoliation of plants, as a renewable source of polyphenols. However, to make the recovery and reuse of agri-food waste and by-products truly effective, green and sustainable models must be developed, as an alternative to conventional methods, aimed at enhancing extraction selectivity, while avoiding excessive consumption of time and solvents. In fact, organic solvents commonly employed in (poly)phenol extraction are currently a matter of concern regarding their potential accumulation in the environment, flammability, and toxicity. Moreover, the obtained crude extracts are generally complex mixtures that require the application of several chromatographic steps to reach an acceptable compound purity. The applied DES extraction, followed by SPE-based flavonol recovery, represents an advantageous strategy, herein favoring the highly selective recovery of quercetin 3-*O*-glucuronide as the main flavonol, together with lower amounts of the 3-*O*-hexosyl derivatives of quercetin itself and kaempferol. Results from the quantitative analysis highlighted its variability, based on the collection time and also on the height of the harvested leaves on the plant. In particular, for upper and middle leaves the content increased from July until early October, when it reached its maximum, whereas the trend in the lowest leaves was exactly the opposite. This evidence underlined that the influence of abiotic factors, such as exposure to sunlight, which is pivotal in the biosynthesis of such molecules, should be strictly considered for their full recovery. 

The DES-based microextraction strategy and SPE-mediated purification, as well as the joint approach of conventional and advanced analytical techniques, are a valid tool to define a process scale-up leading to the full recovery and reuse of plant resources. 

The search for solutions with low environmental impact are strategies to be contemplated to maximize the obtainment of bioactives, thus providing a concrete response to the current sustainable development objectives, and exploring the waste for a nutraceutical eco-friendly innovation.

## Data Availability

Data are contained within the article and Supplementary Materials.

## Supplementary Materials

The following supporting information can be downloaded at: https://www.mdpi.com/article/10.3390/foods12142646/s1, Figure S1: Representative UV–Vis spectrum of SPE fractions eluted with EtOAc; Figure S2: The fragmentation pathway proposed for quercetin 3-*O*-glucuronide (theoretical *m/z* values are reported below each structure); Figure S3: The formation of aglycone ions and the corresponding radical anions observed for hexosyl derivatives of quercetin and kaempferol; Figure S4: (A) HPLC-UV/DAD chromatogram and UV-DAD spectrum related to quercetin; (B) HPLC-MS chromatogram acquired in MRM mode, choosing the most favorable transition (*m/z* 301 → 151) based on the MS/MS spectrum; (C) quercetin calibration curve.
