# Supplementary material for: Quercetin 3-O-Glucuronide from Aglianico Vine Leaves: A Selective Sustainable Recovery and Accumulation Monitoring
Source: Foods. 2023 Jul 9;12(14):2646. doi: 10.3390/foods12142646 (PMC10378925; doi:10.3390/foods12142646)
Supplement: Supplementary file 1 [file foods-12-02646-s001.zip › foods-2488208-supplementary.pdf]

foods

Supplementary Materials

Quercetin 3-*O*-glucuronide from Aglianico vine leaves: a selective sustainable recovery and accumulation monitoring

Elena Cioffi[1], Lara Comune [2], Simona Piccolella[2,*], Mario Buono[1], and Severina Pacifico[2]

[1] Department of Engineering, University of Campania "L. Vanvitelli", Italy
[2] Department of Environmental, Biological and Pharmaceutical Sciences and Technologies, Technologies, University of Campania "L. Vanvitelli", Italy
*Correspondence: simona.piccolella@unicampania.it

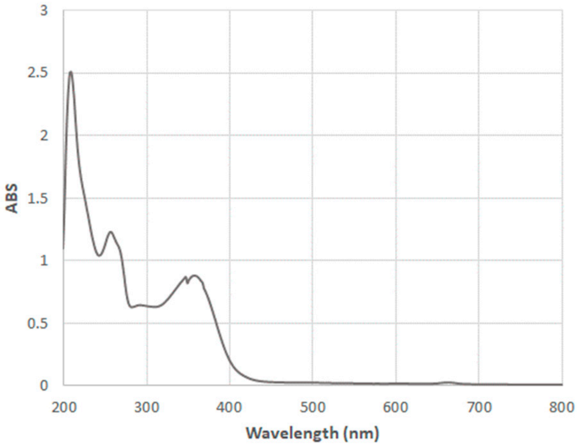

Figure S1. Representative UV-Vis spectrum of SPE fractions eluted with EtOAc.

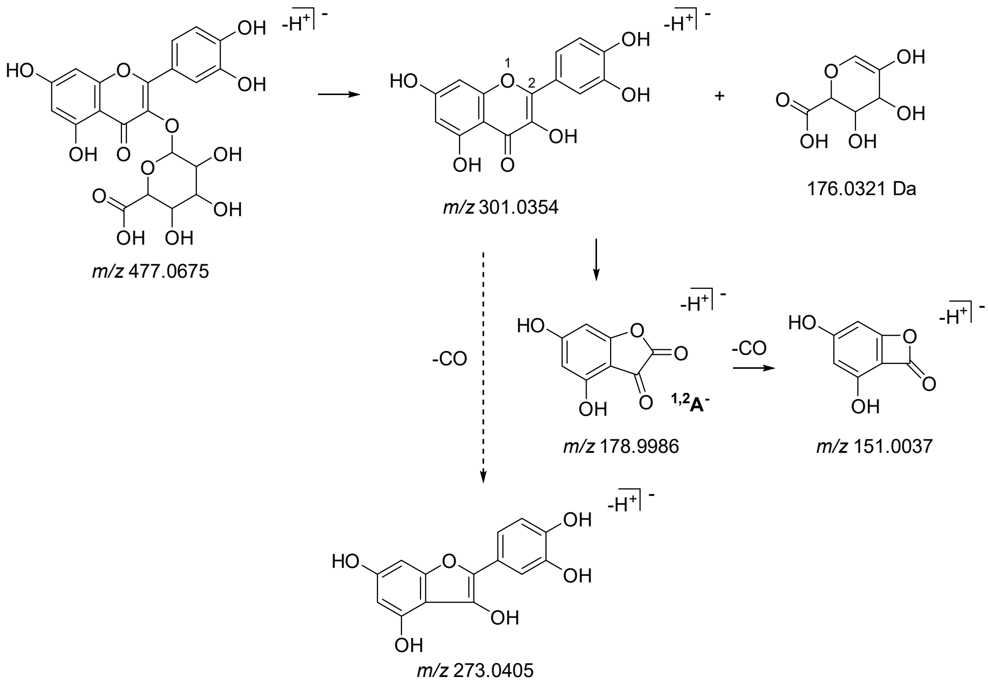

Figure S2. The fragmentation pathway proposed for quercetin 3-*O*-glucuronide (theoretical *m/z* values are reported below each structure).

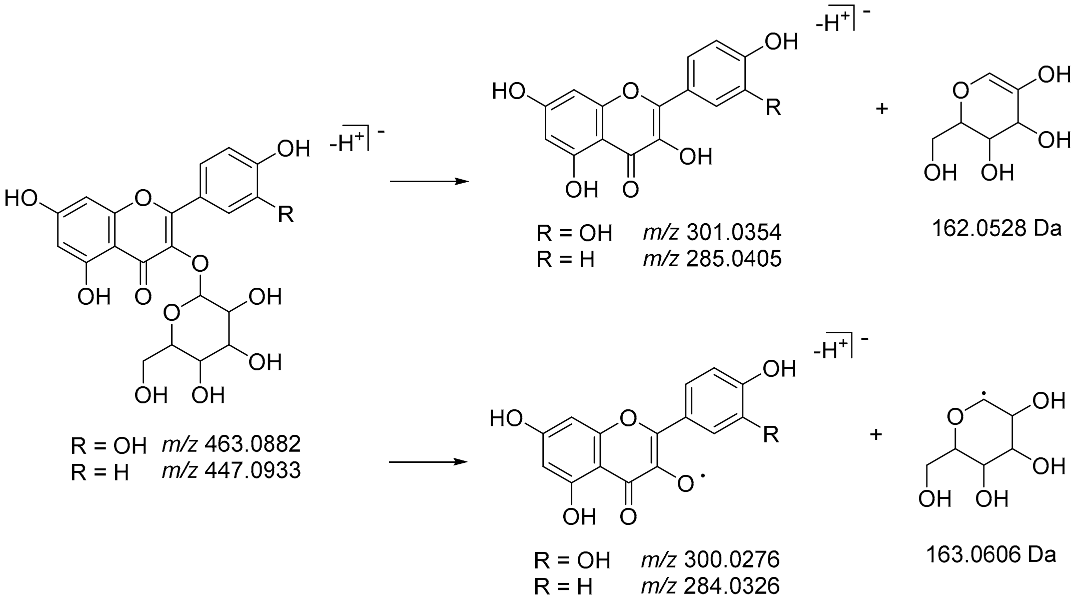

Figure S3. The formation of aglycone ions and the corresponding radical anions observed for hexosyl derivatives of quercetin and kaempferol.

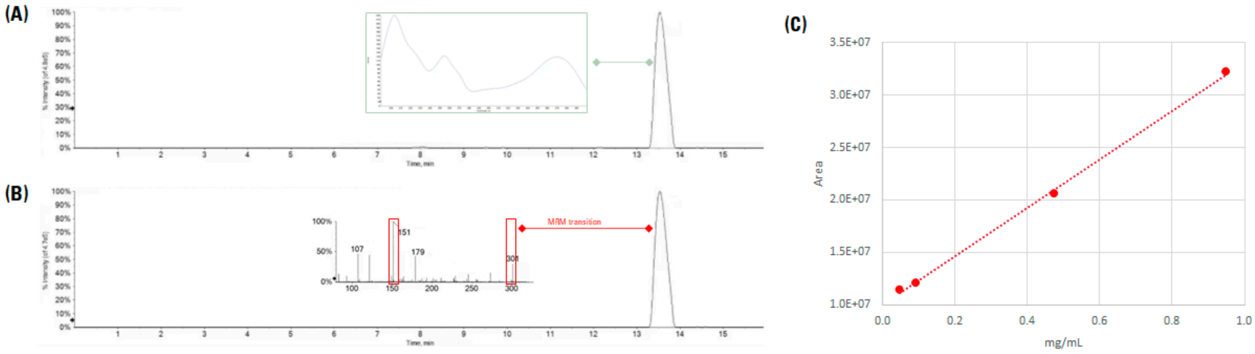

Figure S4. (**A**) HPLC-UV/DAD chromatogram and UV-DAD spectrum related to quercetin; (**B**) HPLC-MS chromatogram acquired in MRM mode, choosing the most favorable transition (*m/z* 301 → 151) based on the MS/MS spectrum; (**C**) quercetin calibration curve.
